# A Preliminary Qualitative Evaluation of an In-home Geriatric Care Elective Experience for Third-year Medical Students

**DOI:** 10.7759/cureus.2415

**Published:** 2018-04-03

**Authors:** Brian J Nagle, Andrea Berry, Laurel Gorman, Mariana Dangiolo

**Affiliations:** 1 Medical Student, UCF College of Medicine, Orlando, USA; 2 Faculty Life, UCF College of Medicine, Orlando, USA; 3 Pharmacology, UCF College of Medicine, Orlando, USA; 4 Department of Internal Medicine, UCF College of Medicine, Orlando, USA

**Keywords:** medical education, geriatrics, in-home care, elective, clerkship

## Abstract

Introduction: The aging population is growing quickly and has a higher prevalence of comorbid and chronic diseases. A majority of this group resides in the home setting. The purpose of this study was to examine the attitudes of third-year medical students following a pilot component of an internal medicine clerkship, consisting of four in-home visits with geriatric patients.

Methods: A qualitative study design, utilizing focus groups, was used to assess general themes in students’ responses regarding their attitudes to geriatrics, the field of geriatrics and the in-home care pilot program.

Results: Twelve students participated in three focus group sessions. Six themes were identified across all focus group sessions. These included 1) distinct advantages to the home setting, 2) more time for relationship building, 3) increased insight to the aging process, 4) increased compassion, 5) suggestions for program improvement, and 6) future quality of care.

Conclusion: The results demonstrate that students found the program to be of value to their medical education. Students developed positive attitudes and compassion for the elderly community. The insight they gained during this program may allow them to understand their role in caring for increasing numbers of aging patients in future populations. Suggestions made by the students showed their perceived value of the program and desire for it to continue for future students. Future studies should utilize validated tools and more longitudinal study designs to assess temporal changes in attitudes.

## Introduction

The age structure of the US is expected to increase drastically by 2050, due to the “baby boomer” population, increased life expectancy, and immigration. The aged population, currently categorized as 65 years and older, will comprise a larger proportion of the total population [1]. The oldest of the aged population (85 years and older) is expected to see the greatest growth, increasing from 14% of the aged population in 2010 to more than 21% by 2050 [2]. The leading causes of mortality and morbidity for this age group differ from those of the younger age groups and continue to change as the life expectancy increases with advances in medicine that decrease the mortality of many leading causes of death, such as heart disease, cancer, pneumonia, and influenza [3].

Another confounding factor for this population is the prevalence of chronic diseases and their impact on the patient’s ability to maintain an independent living situation. Ninety-one percent of older adults have at least one chronic condition, such as diabetes, hypertension, or arthritis, and those who have at least two chronic conditions make up 73% of the aging population [4]. Such chronic diseases are associated with frailty, a decrease in physical reserves that diminish one's ability to adapt to stress, which affects mobility and is predictive of poor health outcomes [5]. This challenging situation along with the myriad of additional issues such as polypharmacy, lack of financial resources, and transportation make it difficult for older adults to remain at home [6-7].

In-home visits by health professionals have been shown to be effective in reducing nursing home placement, functional limitations, and mortality when they have multiple follow-up visits, and specific members of the aging population are appropriately targeted [8]. This highlights the need for healthcare professionals who are well-versed in the aging population’s specific needs as well as the benefit of training professionals to care for patients in their homes.

Despite our knowledge about the benefits of preparing all healthcare professionals to care for the aging population, a gap still exists. Physicians trained in geriatrics are more likely to screen for geriatric syndromes [9], avoid inappropriate medications [10], and demonstrate confidence in caring for the population versus that of primary care or non-geriatric subspecialty trained physicians [11]. To address this knowledge gap and improve confidence, medical schools and residency programs have recently started to implement geriatric experiences as part of their curricula [12].

The purpose of this study was to examine the attitudes of third-year medical students, based on data collected during focus groups, following a pilot component of an internal medicine clerkship, consisting of four in-home visits with geriatric patients. It was expected that the high-quality relationships the students formed with older adults would improve attitudes, build student empathy towards the aging population, and therefore result in greater interest in the field of geriatrics as demonstrated in previous studies [13-16]. It was hypothesized that through the focus groups, third-year medical students would demonstrate positive themes regarding their experience with geriatric patients and the pilot program.

## Materials and methods

Study design

The authors utilized a qualitative approach to explore medical student perceptions about the older adult population, interactions with older adults, and providing care in an in-home setting. Following the students’ participation in a four-visit in-home program, they were asked to participate in semi-structured focus group interviews. Data from the interviews were collected, recorded and analyzed using thematic analysis. Interview questions are provided in Appendix 1.

Participants

Study participants were medical students in their third year who were enrolled in the internal medicine clerkship and completed the in-home geriatric care component. There were 24 students who completed this component of the clerkship. Of the 24 students who participated in the in-home care component, 12 of the students (50%) volunteered to participate in the focus groups. There were seven (58%) female participants and five (42%) males. No data was collected on the students’ age or ethnicity.

Study location

In order to make the focus groups most convenient for students, focus groups were divided into three sessions and scheduled around the students’ clerkship didactic sessions. The in-home component of the internal medicine clerkship took place within the homes of independent senior patients, between the ages of 65 and 100, who receive medical care at a geriatric care clinic. These patients are residents of Orlando, Florida.

Recruitment

Participants were selected from students in their third year of medical school who were enrolled in the internal medicine clerkship and were selected by the clerkship directors to participate in the pilot component. Participation was dependent on a number of factors, such as the availability of independent seniors for in-home visits, the availability of the pilot program’s mentor, and the students’ schedule and clerkship site.

Procedures

In-Home Care Component

The in-home care component covered four main topics that corresponded with a series of four assignments that the students needed to complete at each respective visit and in the following order: 1) taking a medical history using the Comprehensive Geriatric Assessment [17] and performing vital signs screening, 2) performing a fall risk assessment, 3) depression and cognitive assessment, and 4) medication review. For safety in the community setting, students were paired together based on gender, so that each pair of students had at least one female student. Student pairs were assigned to an independent senior who was a patient for the duration of the elective component. A faculty geriatrician provided oversight for students and served as their mentor for the duration of the four-week elective component, as well as the contact for the volunteer patient. Four visits were required over the course of four weeks and students were encouraged to complete one visit per week. The visits occurred in the senior patient’s home setting and were scheduled to be one hour in duration. Students were required to report back to their assigned mentor after each visit in order to ensure suitable patient interventions, a high level of care, and that their assignment for each visit has been successfully completed. Students also met with a pharmacologist and reviewed medication effects and concerns as they conducted the medication review. Additionally, the mentor met with the volunteer patients after the second and fourth week, in order to receive feedback on the students’ performance from the patients. Students were invited to complete two online surveys before and after the four-week component to be examined in separate studies. These included a pharmacology quiz pertaining to the medication review of the in-home care component and the UCLA Geriatric Attitudes Scale [18].

Focus Groups

The questions for the focus groups were developed from answers obtained from the UCLA Geriatric Attitudes Scale with general trends seen with the aggregate data. Participation in the post-experience Geriatrics Attitudes Scale was small and incomplete (n=6), limiting our ability to analyze and present the survey data in this preliminary study. However, a crude analysis was sufficient to develop targeted questions for the quantitative focus group component. These questions can be found in Table 1.

Students invited to participate in the focus group were notified that they would receive a small incentive for participation and that their responses would be kept both anonymous and confidential. Focus groups were conducted in a classroom setting. Sessions were recorded on a password-protected device for data analysis and it was explained to students that after analysis the recordings would be deleted.

Each session lasted between 30 and 45 minutes. Students in each session were all asked the same questions by the same facilitator. In addition to the audio-recording, notes were taken by the investigator. Depending on the pace of the focus group and the amount of time that students had to answer questions, some questions were not asked at every group session or answered by every student. Students were frequently asked to elaborate to ensure that they adequately conveyed their thoughts. If it was not clear what the student was saying, clarification was asked by the investigator for the student to ensure a correct understanding of their answers.

Instruments

UCLA Geriatric Attitudes Scale

The UCLA Geriatric Attitudes Scale is a validated scale, consisting of fourteen statements each requiring a rating using a 5-point Likert scale, ranging from 1 (“Strongly Disagree”) to 5 (“Strongly Agree”). A “Neutral” response is represented by a rating of 3. Five of the statements are worded positively and the remaining nine are worded negatively, and subsequently reversed for scoring purposes. The internal consistency reliability as measured with Cronbach’s alpha was found to be 0.76. Cross-validation coefficient alpha was found to be 0.75. Construct validity was measured by comparison to subscales of Maxwell-Sullivan (“Too Much Time to Care” and “No Benefit of Treatment”) and found to have a significant correlation of 0.58. Validity was also supported by known-groups validity; geriatric faculty and fellows showed significantly higher scores than residents in their first or second year, although the instrument was not validated with undergraduate medical students. The validation study did not show any floor or ceiling effects and the instrument was shown to have sensitivity to change [18].

Data analysis plan

Qualitative analysis followed a strategy of three main steps: 1) description of what was said, 2) categorizing descriptions, and 3) thematic development through the synthesis of categories. Notes taken by the investigator were entered into a spreadsheet with major points and direct quotes taken during the focus groups separated by each question. The recordings were then reviewed and major points and quotes that were missed during note taking were added to the corresponding question. One to three words were used to categorize these major points and quotes in adjacent cells. After these were all categorized, categories were compiled to look for overarching categories and general themes.

## Results

Qualitative data analysis revealed six major themes that spanned the three focus groups. These themes referred not only to experience that the students gained from their participation in the program, but also their evaluation of the program and its role in their medical education. Major themes and students’ noted advantages and concerns are summarized in Table 1.

**Table 1:** Major Themes and Noted Advantages and Concerns

Table 1		
Major Themes and Noted Advantages and Concerns		
Themes	Noted advantages	Noted concerns
Patient home-setting	More complete medication & supplement review	Fewer resources (e.g., medical equipment, medications, lab results) than clinical setting
	More time for patient to prepare	
	More time with the patient	
	Slower pace allowed more thorough discussion	Less formal/professional than clinical setting
	Patient “in their natural setting”	
	Patient “more comfortable in their space”	
Relationship building	More time to build relationship and get “the whole story”	Visit could be time-consuming
	Better communication - more time to listen to patient	
	More time to ensure understanding, write out clear instructions	
	Learning the right questions to ask	
Insight into the aging process	Obtain “a better picture of what their life is like” than if in office	Seeing patients at their best in home setting
	Enhanced ability to access patient environmental risks (falls in environment)	
	Awareness of finances, economic issues	
	Greater ability to access quality of life issues like independence and loneliness	
	Appreciation for how elderly manage complex polypharmacy issues	
	Greater insight to social issues and support	
Compassion	Gained awareness of importance of listening	Expressed concerns about society not understanding barriers elderly face
	Improved empathy for their concerns with medications and lifestyle	Elderly being marginalized in medical care
	Improved patience	Life expectancy increasing but society needs better understanding of how to manage elder care
	Insight into the importance of spending time with elderly patients	
Program design	Rewarding program	Patients independent & self-sufficient; need for more diverse sample of elderly
	Patients were well selected – diverse but open patient group	Need for more time allotted for the visits
	Enjoyed working in pairs of two	Would like to see patients from less privileged backgrounds to better understand wider range of elderly issues (compliance, medication affordability, etc)
	Workbook helpful	Geographical issues (e.g., transportation issues in getting to home visit sites)
	“Continuity” of care – saw same patient for return visit	
Future quality of care	Better insight in geriatrics as a career choice	Feasibility of home-visits due to time and geographical constraints as physician
	More confidence in working with elderly patients	
	Improved understanding of elderly issues would impact future care of elderly patients regardless of specialty	
	Better grasp of “what questions” to ask elderly patients	

Theme 1: The home setting’s distinct advantages and disadvantages

Students noted several advantages to the home setting compared to the clinical setting. It was easier to complete a more thorough review of the patients’ medications, they were able to see where the patient kept their medications and also what supplements or herbal remedies they may have been taking in addition. Students also noted that their patient was more comfortable “in their space” and that it was advantageous “seeing them in their natural setting.” One student also felt that the home visit allowed the patient to prepare their medical history better for them. Most of all, students consistently felt that the home setting provided them an opportunity to spend more time with their patient. In fact, although the visit was only scheduled for an hour, most students stayed with their patient longer than the one hour that was assigned. This increased amount of time with the patient provided the students a greater exposure to the geriatric patient, insight into the geriatric experience, and an opportunity to work on relationship building with the geriatric patient. These will be discussed in later themes.

This is not to say that the in-home setting was without challenges. Students felt that there were fewer tools at their disposal than would be found in the clinical setting, making it harder to accomplish the more clinical aspects of care. One student felt that in the clinical setting you have greater access to lab and test results, while others also admitted that there were fewer resources such as medical equipment and medications. Professionalism was brought up by another student, as they felt that it was easier to maintain professionalism in the clinical setting, but in the home setting they had “to stay aware of professional boundaries.”

Theme 2: More time to enhance relationship building and communication skills

All of the students were in agreement that they were able to build strong relationships with their patients due to their ability to spend more time with them. Most students agreed that they did not believe it was the setting that enabled them to build a stronger relationship with the patients, but the amount of time they were able to spend with them. The student workbook served as a useful guide, as one student felt that they started with that, but then were able to go much more in-depth. They said that it allowed them to get into deep conversations with the patients regarding many aspects of their lives and talk about much more than just their medical concerns and history. One student said that her partner and her “got into really personal stuff” with their patient, which they did not feel they would have been able to do in the clinical setting. “You need the whole story, which you cannot get in 15 minutes,” one student stated, referring to the complexity of patients who have accumulated decades of medical history. Another student felt that seeing them once a week allowed them to build a much better rapport. However, students were also aware that this may not be practical for the medical practice. One student stated that spending over an hour with a patient was not a “luxury” that doctors could afford due to limited health care resources. However, the point was also brought up that this could also be seen as a way to stress the importance of interdisciplinary teams and interprofessional collaboration. Regardless, students did agree that it was a good learning tool for them as medical students and allowed them to practice their communication and rapport building.

Communication skills with this population were also addressed, as students felt that they learned how to better connect with older adults. “It takes no time to have a quick conversation,” said one student, who felt that sometimes all some patients need is for someone to listen to them. Another student said that she learned the importance of ensuring patients understand the care plan and writing instructions down for them, so that they have something they can take home that makes clear the instructions you discussed in the clinic. Students also discussed that by having more conversations with people they were better able to redirect conversations to help with time management, but also the value of sometimes getting away from the chief complaint to see the patient more holistically. Another student mentioned that knowing the right questions to ask also helped with time management to make conversations more efficient, because in the clinical setting, as mentioned before, patient visits must be more efficient due to short times.

Theme 3: Students’ insight into the geriatric experience

Students overwhelmingly felt that they walked away from the experience with much more insight into the geriatric patients’ life. Through their exposure with the patient they said that they learned about many different aspects of their lives. For starters, many of them noted the value of being able to see firsthand the patients’ home environment. One example that was provided regarded their assessment of the home for fall risk and that it was much more valuable to be there in person to objectively evaluate it than being in the office and asking the patient subjectively. One student said that being inside their home provided “a better picture of what their life is like.”

The patients’ daily activities and ability to function independently was also addressed as something the student gained insight into. In reference to one student learning from his patient that they had not moved a piece of furniture, because they were not physically able to do so, they stated to the focus group, “That is a struggle I have no experience with right there.” Issues related to driving, finances, and social support were discussed. One student stated that they had “more knowledge about financial issues and a better understanding of financial constraints.” “They are on a really tight budget,” another student stated. One focus group discussed scope of practice with regards to finances and the value of interprofessionalism to aid in the areas of life outside of health care that can have a significant impact on the patients’ health. Students learned about patients’ friends who were isolated and lonely and their patients’ views on being isolated. One student summed it up by saying, “When you see them at home, you get a better picture of what their life is like outside the visit, their list of medical problems, and their medications. You have an understanding.  Do they live alone? How many people are living with them? Do they have help? If they have a diagnosis, are they going to go home and have to care for themselves? Are they going to go home to four kids, eight grandkids, and their spouse?” 

When it came to the patients’ health care and quality of life, students re-conceptualized previous mental models. Regarding their health care, topics discussed dealt with access to care, knowledge of their medical history, and medication regimens. “The complexity of the elderly patient is mind-blowing,” one student stated. Another student appreciated the difficulty that the elderly can have with regards to navigating the healthcare system and said they helped to schedule some of the patients’ appointments. Some of the students discussed the need to modify the health care system to make it more accessible and “decrease the number of hoops to jump through to encourage personal responsibility.” One student admitted that their patient forgot certain parts of their history, but the student also recognized that it must be “difficult to remember everything in a long lifetime.” Another student said, “They want to be healthy; they don’t’ think they will live forever like young people.” Students also discussed their patients’ appreciation of their care and desire to be a good patient. Medications was another topic covered, as students found that they were surprised at how organized and systematic the patients were about their medications. “Her system was thought through very well. I have no doubt she was complying,” one student said.

Finally, students also expressed gaining some insight into the elderly patients’ quality of life. Students discussed how physically and socially active their patients were and that they had such a positive outlook. One student felt that this showed them that there is “hope for elderly patients” and another student said that they felt that getting a different perspective on the elderly maintaining their quality of life was important. Overall, students expressed the significance of seeing a patient at their best and that it was a “good example of overcoming obstacles.”

Theme 4: An increase in students’ compassion

Students also showed a great deal of compassion and understanding toward the elderly. They stated that they felt they had more patience after this experience and that sometimes the patients just needed “someone to talk to about their health; someone who can appreciate it.” One student is still in contact with her patient through email. Students who stayed longer than their assigned hour said that they did not mind giving up another hour or two of their time and recognized that these patients got attention in the home visits that they would not receive in the clinical setting.

One student stated, “Without them, we wouldn’t even be there. Sacrifices that they have made have given us opportunities.” This sentiment that society could do better to care for its elderly population was shared over the three focus groups. Students described them as on “the margins of society” and “forgotten.” Students discussed how historically it was the families who cared for their elders and that there were still other cultures around the world, where the elderly were valued, not marginalized. “We owe it to them to at least offer some level of support,” one student stated. Another student who felt that there was still some level of personal responsibility on the patient, also said that they realized the difficulties they face and agreed with their classmates that steps should be made to decrease some of these barriers. Another student was torn between the two views, “On one hand you set yourself up to be successful and taken care of at the end, but there’s another component that you have given and should also receive.” Still, others discussed the ramifications of the increasing life expectancy: “People are living longer and longer, so their retirement, they didn’t plan to live for another 30 years.”

Theme 5: Program evaluation

Students found the in-home care program to be rewarding and gave praise for various aspects of its design. Many students felt that the patients were selected well, as they were very open and welcoming, and did not seem to mind when the students were required to “poke and prod to find out new information each visit.” One pair of students felt that they “clicked” with their patient. Other aspects of the program that students liked were the continuity they had with the same patient, the workbook that was provided to them, and that each visit had a specific objective that they were to achieve. One focus group discussed their appreciation of having the students paired together, as they would not have done the program alone.

Although students did have much praise for the program and found it to be very worthwhile, they also voiced their opinions on ways that the program could be improved. Suggestions for improvements that would benefit students, as well as the patients, were discussed. One of the most widely discussed suggestions centered on patient selection. Although many valued the time spent with their patient, they also felt that perhaps their positive experiences were biased by the patient selection process. Students whose patient had been with another patient pair in a more recent session were divided on the benefit to the patient. Some felt that when their patient had already seen a student pair that it “felt a little like time was wasted” and that it was “not worth doing unless it’s a new patient.” Still, another patient pair felt that it depended on the patient, because although their patient had previously seen a student pair, they felt that based on her medical conditions she still benefited greatly from their visits and saw it more as a continuation of her care from the previous group.

Many students said that they would also like to see “the other side” with patients who were “not in ideal conditions” or who were “less educated.” Another student felt that it would be beneficial to see patients who were not as independent, and another suggested seeing two types of patients, both an independent patient and one that would be less independent. Students felt that this would not only benefit their experience with the program and give them more exposure to different types of patients in the home-setting, but that patients who are less independent or less educated would benefit more from the in-home visits. One student felt that it was a program that patients in underserved communities would get the most benefit from.

Increasing the amount of time scheduled for the patient visit was almost universally shared by all of the students. Many felt that the hour that they were scheduled to spend with the patient was not enough, especially in the first visits. Most suggested adding another hour to the scheduled time to reflect a more accurate prediction of the amount of time that the students would spend at the visit. They did not feel that this should be a required amount of time to spend with the patient, but just to let students know beforehand that the visit may take longer than an hour.

Students also weighed in on the feasibility of some of these suggestions. Safety and geographic constraints were two of the biggest limitations that they discussed. While they did feel that underserved communities and patients who were less independent would benefit the most, they acknowledged that there were risks when going into someone’s home and that “not all patients live in a safe environment.” Geographic constraints restricted feasibility as students had to factor in the drive time to the patient’s home. One student said the “drive was terrible” and that it was “hard to plan around.”

Theme 6: Future quality of care

Students reflected on the impact that this experience had on their future career plans. Although no student expressed any desire to pursue a career in geriatrics, a majority did feel that the program would positively affect their career and prepare them for specialties other than geriatrics. One student said that they did not want to be a geriatrician, but said that “it takes a certain person to do it” and that they had a better appreciation of those who pursued geriatrics as a specialty. Another student said, “I am less intimidated to have them as part of my patient population.” Other students expressed an increase in their willingness to work with the aging population in the future. “People are living longer. We will need to work with them,” one student noted, referring to the increasing size of the aging population and the many specialties they will likely encounter.

Across all focus groups, students felt that, while they questioned the feasibility of in-home visits in their career, they came away from the program with a greater appreciation of the kinds of issues that the elderly face in the home setting and that exposure to these issues would impact their clinical encounters with elderly patients. Many students also felt that the insight they gained would aid them in what types of questions to ask this age group. “I know questions to ask more specifically,” said one student. Students agreed that it gave them a more objective and accurate assessment and perspective of the patient’s home life. One student compared this to the well-child visits they were doing as part of the pediatric clerkship. This student expressed that they saw firsthand the importance of addressing issues like fall risk, stating, “It’s worth addressing those things at an annual appointment. There are ways to improve their health other than with medications that you can do in the clinic that I previously didn’t know about.” Other students saw the benefit of witnessing solutions to problems in the home setting that have worked for their patient. “Learning her system, the way she had it set up, was good as far as me as a doctor then suggesting that to another patient,” one student said, referring to their patients’ system for taking medications, “seeing how that can work and somebody can be compliant on that many medications. I do think that you can offer them quite a bit to improve their quality of life and make their last years as good as they possibly could be.”

## Discussion

The main goal of this study was to utilize focus groups to assess third-year medical students’ attitudes of a pilot component of an internal medicine clerkship, consisting of four in-home visits with geriatric patients. Students were asked questions about their impression of the in-home visits and how it affected their view of the aging population, the field of geriatrics, and their future career as physicians.

For several years there have been efforts to increase exposure to the geriatric patient population in undergraduate medical education in the US [19]. Several programs have been developed at different medical schools to facilitate this goal and create innovative ways for students to interact with the aging population during the formative years of their medical career [20-26]. Authenticity has been cited by students as one of the biggest advantages of working with real patients in the community as opposed to standardized patients [27]. Findings in this study are in line with many of the findings from other programs that have brought elderly patients and community members together with students.

The six themes that were found across the focus group sessions demonstrate that the students found this program to be of great value. By entering the patients’ homes and objectively viewing the home setting, students were better able to get a sense of the aging person’s life outside of the clinical setting. Similar findings were found with students who worked in the homes of disabled elderly, increasing the students’ recognition of the importance of the home modifications and social support to the patients’ well-being [20]. Additionally, a similar program increased students’ awareness of the impact that the complexity of the health care system has on elderly patients’ health [28].

Students showed dedication to the program, by not only staying the hour that they were scheduled to visit the patient to complete their session objectives, but frequently staying longer than they were expected. Students used this extra time to develop relationship building skills with their patients. As demonstrated in other studies, this increase in time spent building a rapport with the patient and observing the patients’ surroundings help students to gain a great deal of insight about and compassion for the aging population. [22-24, 28]. The students’ positive attitudes about the elderly influence views of their future career and care for the growing aging population. Like the students in this study, medical students have noted a change in not only knowledge about geriatric care, but also awareness regarding the level of happiness and independence geriatric patients can have in their life despite debilitating disabilities [20, 22]. Patients’ levels of social engagement and physical activity are additional aspects of their quality of life which is surprising for most students [21]. This empathy and relationship building is important, as “emotional content” has been recognized by second-year students to be one of their most deficient areas of training [29]. Suggestions made to improve this program reflect the students’ interest in the program and seeing it continue with the subsequent classes of medical students. By expressing to work with a broader range of patients, students showed an eagerness to explore a wider variety of patients’ home settings. Their acknowledgment of the barriers to implementing such changes is consistent with a previous study, where students recognized the advantages to working with real patients, but also acknowledged the difficulty in recruiting real patients [29].

Despite the promising findings in this qualitative study, there are several limitations. As this was a pilot component of the clerkship, the sample size was limited due to convenience, the number of patients willing to participate, and student availability to participate as students are geographically distributed and have varying schedules. There appeared to be a high degree of agreement across focus groups, which could be attributed to selection bias, as those who had more positive experiences in the program may have been more likely to participate in the focus group. Demographics, such as the students' age or ethnicity, were also not collected, which may play a role in their attitudes toward the geriatric population. A qualitative study design utilizing focus groups was selected for this preliminary study, however, focus groups are susceptible to social desirability bias. Therefore, additional research is indicated utilizing other methods and validated tools, such as the UCLA Geriatric Attitudes Scale, to assess students’ changes in attitudes toward the elderly and the field of geriatrics. [18]. The strategic approach to recruiting patients to the program likely impacted the students’ impression as to whether the encounters were realistic. Such uncertainty may lead them to question their ability to care for the older adult population. Additionally, the educational value of clerkship level students performing medication reviews in the elderly needs to be ascertained with future students, as appropriate prescribing for the elderly is a major patient safety concern [30], and this could be an area for interprofessional development with a pharmacist or pharmacy students. A randomized control trial would be ideal in examining students’ changes in attitudes, and would increase the validity and decrease the potential for social desirability bias, as well as increase the opportunity for additional students to participate.

## Conclusions

Qualitative results of this study of the pilot geriatric in-home care component for third-year medical students show promising results. Major themes from the focus group were advantages of the home setting compared to the clinical setting, increased insight into the aging process, more time for relationship building with the patient, increased sense of compassion for the geriatric population, suggestions to improve program design, and the perceived impact it will have on their future career in various specialties. More rigorous quantitative prospective studies should be used to examine the effect that this program has on their feelings toward the geriatric population, using validated instruments to look for significant changes. Additionally, tracking their scores over time to look for changes further in their fourth-year of medical school and into residency would also provide insight into if and how these feelings change throughout their training.

## Appendices

Semi-structured questions for student focus groups of the in-home care vomponent

1) What kind of preconceived notions did you have going into the in-home care setting?

2) How do you feel about the elderly patient population now? How has this influenced your career choice?

3) What are some things you took away from the in-home care component you participated in? Probes: continuity of care, access to care, real-world assessments/things you wouldn’t see in the clinic, opportunities for patient education?

4) Having interacted with the elderly in two different environments, in-home care and in the clinical setting, how were these two experiences different for you? Advantages? Disadvantages?

5) Having spent four visits with the same patient, how would you describe your relationship with that person at the end of the program? How did that relationship change over the course of the visits? Positive? Get along? Negative?

6) Through some of the survey data, we noticed that there were some changes in some questions from more positive responses in the pre-surveys to less positive attitudes in the post-surveys, so I wanted to discuss those a little bit. [Confirm before each that they feel this way]

For starters, fewer people were inclined to choose to see older patients after completion of the in-home care component. What factors do you think would explain this?

Fewer people felt it was society’s responsibility to care for the elderly. What kind of factors would lead to this feeling?

Additionally, more people felt that the elderly were disorganized and confused. What kinds of factors would have made people more likely to feel this way?

A majority of people taking the post-survey did not feel that more attention and sympathy should be given to the elderly. Why do you think this is?

7) There were some positive differences seen as well, that I would also like to touch on. [Confirm before each that they feel this way]

For starters, a majority felt afterward that the elderly were more pleasant. What kinds of experiences influenced this change?

More people disagreed with the statement that money should be reallocated from Medicare to AID and pediatric diseases. Were there things that you witnessed or experienced that changed your feelings on this?

More people also felt afterward that elderly patients were more appreciative. Again, what experiences did you have that might explain this shift?

A majority also disagreed with the statement that taking a medical history from an elderly patient was an ordeal. What were your thoughts before and how did they change?

Finally, a majority at the end did feel that the elderly did contribute to society and also to their health care payments. Did you witness anything in particular that would explain this shift in attitude?

8) How many of you would say that you had a positive relationship with at least one elderly person before medical school?          

What kinds of relationships were these? In your family? Neighbors? Work? Volunteering?

How do you think these relationships affected your mindset about the in-home care?
